# Crystal structure of 4,4′,4′′-(1,3,5-triazine-2,4,6-tri­yl)tripyridinium trichloride 2.5-hydrate

**DOI:** 10.1107/S2056989015018125

**Published:** 2015-10-17

**Authors:** Bo-Kai Ling, Xiao-Long Feng, Yang Li, Tian-Gang Luan

**Affiliations:** a School of Marine Science, Sun Yat-Sen University, Guangzhou 510275, People’s Republic of China; b Instrumental Analysis and Research Center, Sun Yat-Sen University, Guangzhou 510275, People’s Republic of China

**Keywords:** crystal structure, 1,3,5-triazine, trichloride, hydrogen bonding, π–π inter­actions

## Abstract

The asymmetric unit of the title compound, C_18_H_15_N_6_^3+^·3Cl^−^·2.5H_2_O, contains two independent (1,3,5-triazine-2,4,6-tri­yl)tripyridinium cations. Both cations are approximately planar, the r.m.s. deviations of fitted non-H atoms being 0.045 and 0.051 Å. In the crystal, extensive O—H⋯Cl, O—H⋯O, N—H⋯Cl and N—H⋯O hydrogen bonds and weak C—H⋯Cl and C—H⋯O inter­actions link the organic cations, Cl^−^ anions and water mol­ecules into a three-dimensional supra­molecular architecture. π–π stacking between the pyridine rings of adjacent cations is also observed, the centroid-to-centroid distance being 3.7578 (8) Å.

## Related literature   

For applications of 2,4,6-tris­(pyridin-4-yl)-1,3,5-triazine, see: Yoshizawa *et al.* (2006 ▸); Inokuma *et al.* (2011 ▸, 2013 ▸). For the crystal structure of 2,4,6-tris­(pyridin-4-yl)-1,3,5-triazine (TPT), see: Janczak *et al.* (2003 ▸). For the crystal structure of (1,3,5-triazine-2,4,6-tri­yl)tripyridinium nitrate, see: Zhu *et al.* (2007 ▸).
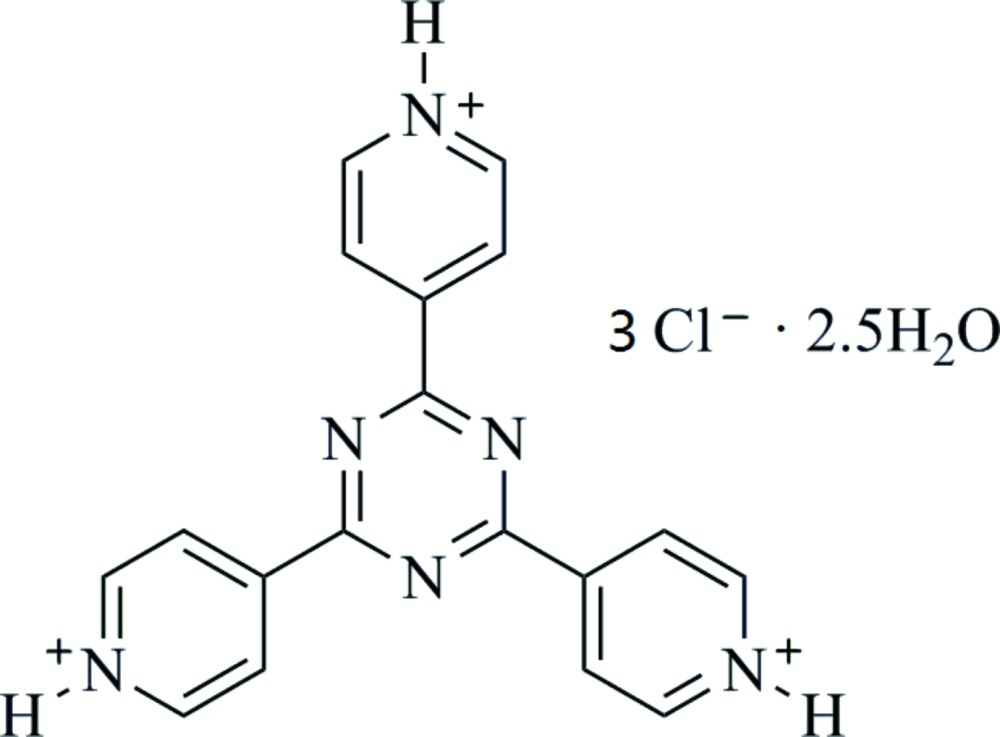


## Experimental   

### Crystal data  

2C_18_H_15_N_6_^3+^·6Cl^−^·5H_2_O
*M* *_r_* = 933.50
Monoclinic 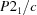

*a* = 10.6042 (1) Å
*b* = 14.6447 (1) Å
*c* = 27.7906 (3) Å
β = 98.310 (1)°
*V* = 4270.44 (7) Å^3^
*Z* = 4
Cu *K*α radiation
μ = 4.15 mm^−1^
*T* = 150 K
0.50 × 0.20 × 0.10 mm

### Data collection  

Agilent Xcalibur Atlas Gemini ultra diffractometer
Absorption correction: multi-scan (*CrysAlis PRO*; Agilent, 2014 ▸) *T*_min_ = 0.575, *T*_max_ = 1.000
27170 measured reflections
7278 independent reflections
6654 reflections with *I* > 2σ(*I*)
*R*_int_ = 0.020

### Refinement  

*R*[*F* ^2^ > 2σ(*F* ^2^)] = 0.026
*wR*(*F* ^2^) = 0.076
*S* = 1.05
7278 reflections
572 parameters
H atoms treated by a mixture of independent and constrained refinement
Δρ_max_ = 0.26 e Å^−3^
Δρ_min_ = −0.25 e Å^−3^

### 

Data collection: *CrysAlis PRO* (Agilent, 2014 ▸); cell refinement: *CrysAlis PRO*; data reduction: *CrysAlis PRO*; program(s) used to solve structure: *SHELXS97* (Sheldrick, 2008 ▸); program(s) used to refine structure: *SHELXL2014* (Sheldrick, 2015 ▸); molecular graphics: *ORTEP-3 for Windows* (Farrugia, 2012 ▸); software used to prepare material for publication: *WinGX* (Farrugia, 2012 ▸).

## Supplementary Material

Crystal structure: contains datablock(s) I, global. DOI: 10.1107/S2056989015018125/xu5874sup1.cif

Structure factors: contains datablock(s) I. DOI: 10.1107/S2056989015018125/xu5874Isup2.hkl

Supporting information file. DOI: 10.1107/S2056989015018125/xu5874Isup3.cml

**Fig. 1.**
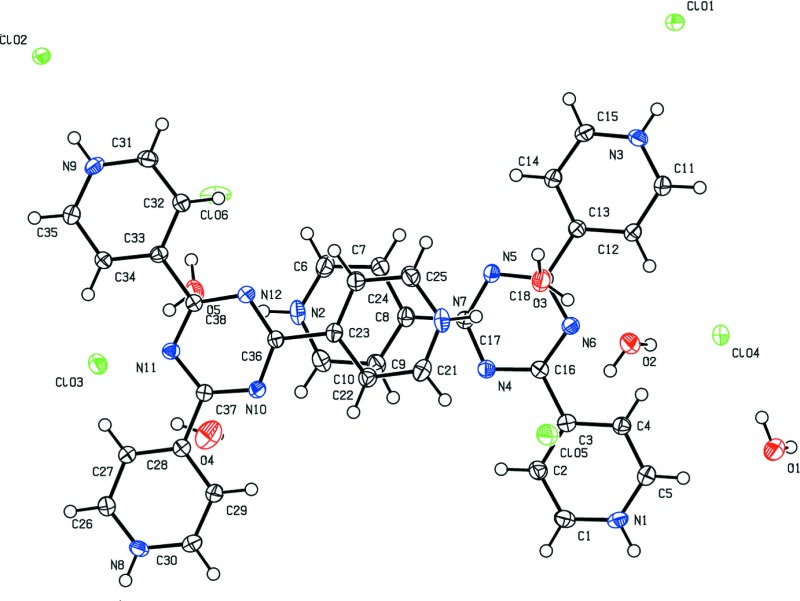
The molecular structure of the title compound, showing the atom labeling. Displacement ellipsoids are drawn at the 50% probability level.

**Fig. 2.**
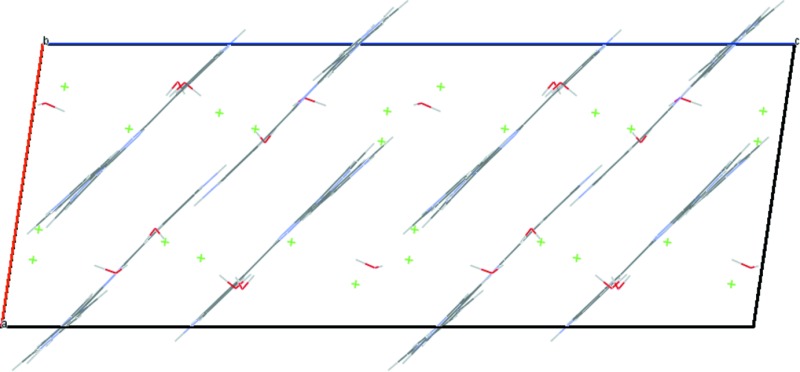
The crystal packing of the title compound viewed along the b axis. Colour key: red indicates oxygen and green chlorine.

. DOI: 10.1107/S2056989015018125/xu5874fig1.tif
The mol­ecular structure of the title compound, showing the atom labeling. Displacement ellipsoids are drawn at the 50% probability level.

b . DOI: 10.1107/S2056989015018125/xu5874fig2.tif
The crystal packing of the title compound viewed along the *b* axis. Colour key: red indicates oxygen and green chlorine.

CCDC reference: 1427933

Additional supporting information:  crystallographic information; 3D view; checkCIF report

## Figures and Tables

**Table 1:** Hydrogen-bond geometry (, )

*D*H*A*	*D*H	H*A*	*D* *A*	*D*H*A*
O1H1*A*Cl03^i^	0.77(2)	2.47(2)	3.2302(15)	171(2)
O1H1*B*Cl04	0.87(2)	2.32(2)	3.1856(14)	175(2)
O2H2*A*Cl04	0.86(2)	2.28(2)	3.1124(15)	163.2(18)
O2H2*B*Cl05	0.84(2)	2.22(2)	3.0515(13)	170(2)
O3H3*A*Cl06^ii^	0.84(2)	2.22(2)	3.0379(13)	164.5(18)
O3H3*B*O2	0.84(2)	1.91(2)	2.7426(17)	176(2)
O4H4*A*Cl05^iii^	0.78(2)	2.36(2)	3.1356(15)	172(2)
O4H4*B*Cl03	0.83(3)	2.47(3)	3.2626(14)	162(3)
O5H5*A*Cl06	0.92(2)	2.12(2)	2.9973(12)	157.4(17)
O5H5*B*Cl03	0.81(2)	2.24(2)	3.0466(12)	173(2)
N1H01Cl01^iv^	0.86	2.24	3.0678(12)	161
N2H02O5	0.86	1.77	2.5985(16)	162
N3H03Cl01	0.86	2.23	3.0405(12)	158
N7H07O3	0.86	1.84	2.6472(16)	155
N8H08Cl02^iv^	0.86	2.25	3.0732(12)	159
N9H09Cl02	0.86	2.19	3.0337(12)	166
C1H1Cl03^v^	0.93	2.57	3.4995(15)	174
C4H4Cl04	0.93	2.61	3.4871(14)	157
C5H5O1	0.93	2.38	3.2614(19)	158
C9H9Cl06^v^	0.93	2.70	3.4071(15)	134
C10H10O4	0.93	2.54	3.370(2)	148
C11H11Cl02^vi^	0.93	2.61	3.5114(15)	163
C12H12Cl04	0.93	2.64	3.4992(15)	154
C15H15Cl03^vii^	0.93	2.54	3.3157(16)	141
C21H21Cl05	0.93	2.74	3.5424(15)	146
C22H22Cl06^v^	0.93	2.72	3.3883(15)	130
C24H24Cl05^viii^	0.93	2.63	3.4519(15)	147
C26H26Cl04^ix^	0.93	2.53	3.3669(15)	149
C30H30O2^x^	0.93	2.31	3.2331(19)	174
C31H31O2^viii^	0.93	2.50	3.3320(19)	149
C34H34O1^ix^	0.93	2.50	3.3706(19)	156
C35H35Cl01^xi^	0.93	2.72	3.6254(14)	166

Symmetry codes: (i) 
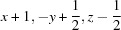
; (ii) 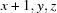; (iii) 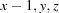; (iv) 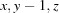; (v) 
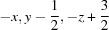
; (vi) 
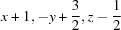
; (vii) 
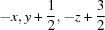
; (viii) 
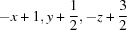
; (ix) 
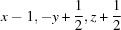
; (x) 
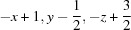
; (xi) 
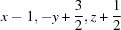
.

## supplementary crystallographic information

### S1. Comment

2,4,6-Tris(4-pyridyl)-1,3,5-triazine (TPT), as a planar tridentate ligand for MOFs (metal-organic frameworks), has been designed for some useful crystals by reactions with metal ions. Due to its special triazine *π–π* inter­action, triangular plane geometry and tridentate N atoms coordinate, these crystals remarkable applications were discovered gradually such as molecular flask (Yoshizawa & Fujita, 2006; Inokuma & Fujita, 2011) and X-ray single-crystal diffraction carrier (Inokuma & Fujita, 2013). The crystal structure of neutral tpt was reported by (Janczak & Kubiak, 2003). The nitrate salt of TPT was published by Zhu (Zhu *et al.* 2007).

The crystal has a well layered form through *π-π* inter­action and Hydrogen Bonds which is analogous to pure TPT crystal (Janczak & Kubiak, 2003). In the crystal, every pyridine has protonized. H_3_TPT, Cl^-^ and H_2_O pack in a layer through ionic bonding and hydrogen-bonding.

### S2. Synthesis and crystallization

Excess hydro­chloric acid (2 mL) was added in pure TPT (93mg, 0.3mmol) in a 20 mL scintillation vial. With the dropwise addition of hydro­chloric acid, solution was clear gradually. Then the mixture was put in an oven at 393K for 10h. The colourless crystal will be found.

### S3. Refinement

All H atoms for C and N atoms were geometrically fixed and allowed to ride on their parent C and N atoms, with C–H = 0.93 Å, N–H = 0.86 Å) and with *U*_iso_(H) = 1.2*U*_eq_(C), *U*_iso_(H) = 1.2*U*_eq_(N). H atoms belonging to H_2_O groups were located in difference Fourier maps and refined isotropically.

### Crystal data

**Table:**

2C_18_H_15_N_6_^3+^·6Cl^−^·5H_2_O	*F*(000) = 1928
*M**_r_* = 933.50	*D*_x_ = 1.452 Mg m^−^^3^
Monoclinic, *P*2_1_/*c*	Cu *K*α radiation, λ = 1.54184 Å
*a* = 10.6042 (1) Å	Cell parameters from 15012 reflections
*b* = 14.6447 (1) Å	θ = 3.0–65.5°
*c* = 27.7906 (3) Å	µ = 4.15 mm^−^^1^
β = 98.310 (1)°	*T* = 150 K
*V* = 4270.44 (7) Å^3^	Rod, colourless
*Z* = 4	0.50 × 0.20 × 0.10 mm

### Data collection

**Table:**

Agilent Xcalibur Atlas Gemini ultra diffractometer	6654 reflections with *I* > 2σ(*I*)
Detector resolution: 10.5058 pixels mm^-1^	*R*_int_ = 0.020
ω scans	θ_max_ = 65.6°, θ_min_ = 3.2°
Absorption correction: multi-scan (*CrysAlis PRO*; Agilent, 2014)	*h* = −12→12
*T*_min_ = 0.575, *T*_max_ = 1.000	*k* = −11→16
27170 measured reflections	*l* = −32→31
7278 independent reflections	

### Refinement

**Table:**

Refinement on *F*^2^	0 restraints
Least-squares matrix: full	Hydrogen site location: inferred from neighbouring sites
*R*[*F*^2^ > 2σ(*F*^2^)] = 0.026	H atoms treated by a mixture of independent and constrained refinement
*wR*(*F*^2^) = 0.076	*w* = 1/[σ^2^(*F*_o_^2^) + (0.0455*P*)^2^ + 1.0459*P*] where *P* = (*F*_o_^2^ + 2*F*_c_^2^)/3
*S* = 1.05	(Δ/σ)_max_ = 0.002
7278 reflections	Δρ_max_ = 0.26 e Å^−^^3^
572 parameters	Δρ_min_ = −0.25 e Å^−^^3^

### Special details

**Table:**

Geometry. All e.s.d.'s (except the e.s.d. in the dihedral angle between two l.s. planes) are estimated using the full covariance matrix. The cell e.s.d.'s are taken into account individually in the estimation of e.s.d.'s in distances, angles and torsion angles; correlations between e.s.d.'s in cell parameters are only used when they are defined by crystal symmetry. An approximate (isotropic) treatment of cell e.s.d.'s is used for estimating e.s.d.'s involving l.s. planes.

### Fractional atomic coordinates and isotropic or equivalent isotropic displacement parameters (Å^2^)

**Table:**

	*x*	*y*	*z*	*U*_iso_*/*U*_eq_	
Cl01	0.65529 (3)	0.87819 (2)	0.53046 (2)	0.02080 (9)	
Cl02	−0.15165 (3)	0.86725 (2)	0.96259 (2)	0.02038 (9)	
Cl03	−0.30029 (3)	0.33680 (2)	0.86906 (2)	0.02704 (9)	
Cl04	0.76231 (3)	0.34758 (2)	0.52883 (2)	0.02860 (10)	
Cl05	0.69917 (4)	0.19615 (3)	0.70100 (2)	0.03228 (10)	
Cl06	−0.24393 (4)	0.61127 (3)	0.75183 (2)	0.03989 (12)	
O5	−0.13385 (10)	0.45402 (8)	0.81302 (4)	0.0293 (2)	
O3	0.65114 (11)	0.45593 (9)	0.68539 (4)	0.0292 (2)	
O2	0.80923 (11)	0.34430 (8)	0.64199 (5)	0.0308 (2)	
O1	0.79029 (12)	0.14892 (9)	0.48489 (5)	0.0349 (3)	
N11	0.05285 (10)	0.36030 (8)	0.90020 (4)	0.0181 (2)	
N10	0.20086 (10)	0.29011 (7)	0.85541 (4)	0.0189 (2)	
N6	0.46303 (11)	0.37119 (8)	0.59643 (4)	0.0193 (2)	
N5	0.33219 (11)	0.46467 (8)	0.63822 (4)	0.0199 (2)	
O4	−0.13673 (13)	0.20960 (9)	0.80408 (5)	0.0434 (3)	
N4	0.32306 (11)	0.30340 (8)	0.64532 (4)	0.0204 (2)	
N12	0.18691 (10)	0.45145 (8)	0.85844 (4)	0.0190 (2)	
N9	−0.06099 (11)	0.67836 (8)	0.93866 (4)	0.0226 (3)	
H09	−0.0926	0.7271	0.9494	0.027*	
N3	0.57300 (11)	0.68432 (8)	0.54975 (4)	0.0229 (3)	
H03	0.6040	0.7318	0.5375	0.027*	
N1	0.52800 (11)	0.03964 (8)	0.57480 (4)	0.0224 (3)	
H01	0.5519	−0.0134	0.5663	0.027*	
N8	−0.00911 (11)	0.02831 (8)	0.92431 (4)	0.0225 (3)	
H08	−0.0319	−0.0247	0.9332	0.027*	
N7	0.51720 (11)	0.39554 (9)	0.75219 (4)	0.0251 (3)	
H07	0.5727	0.4001	0.7326	0.030*	
N2	0.00613 (11)	0.41058 (9)	0.74708 (4)	0.0268 (3)	
H02	−0.0498	0.4151	0.7665	0.032*	
C16	0.41129 (12)	0.29914 (9)	0.61556 (5)	0.0181 (3)	
C36	0.23658 (12)	0.37382 (9)	0.84431 (5)	0.0173 (3)	
C37	0.10782 (12)	0.28751 (9)	0.88325 (5)	0.0164 (3)	
C17	0.28607 (13)	0.38778 (9)	0.65473 (5)	0.0186 (3)	
C38	0.09613 (12)	0.44037 (9)	0.88672 (5)	0.0171 (3)	
C23	0.33836 (13)	0.38155 (9)	0.81286 (5)	0.0191 (3)	
C18	0.42064 (12)	0.45195 (9)	0.60929 (5)	0.0183 (3)	
C33	0.04010 (12)	0.52483 (9)	0.90481 (5)	0.0181 (3)	
C28	0.06487 (12)	0.19579 (9)	0.89691 (5)	0.0179 (3)	
C34	−0.04614 (13)	0.51835 (9)	0.93800 (5)	0.0206 (3)	
H34	−0.0701	0.4616	0.9486	0.025*	
C8	0.18526 (13)	0.39663 (10)	0.68655 (5)	0.0203 (3)	
C3	0.45303 (12)	0.20713 (9)	0.60187 (5)	0.0190 (3)	
C12	0.56434 (13)	0.52499 (10)	0.55698 (5)	0.0211 (3)	
H12	0.5915	0.4673	0.5490	0.025*	
C22	0.39093 (13)	0.30344 (10)	0.79539 (5)	0.0221 (3)	
H22	0.3655	0.2459	0.8043	0.027*	
C4	0.54831 (13)	0.19906 (9)	0.57262 (5)	0.0204 (3)	
H4	0.5869	0.2509	0.5620	0.025*	
C27	−0.02943 (13)	0.18762 (9)	0.92639 (5)	0.0214 (3)	
H27	−0.0680	0.2394	0.9371	0.026*	
C13	0.47470 (12)	0.53481 (9)	0.58865 (5)	0.0189 (3)	
C2	0.39638 (14)	0.12860 (10)	0.61731 (5)	0.0232 (3)	
H2	0.3332	0.1329	0.6372	0.028*	
C9	0.13433 (13)	0.31852 (10)	0.70484 (5)	0.0236 (3)	
H9	0.1610	0.2609	0.6965	0.028*	
C7	0.14280 (14)	0.48231 (10)	0.69901 (5)	0.0250 (3)	
H7	0.1751	0.5352	0.6868	0.030*	
C31	0.02052 (14)	0.68671 (10)	0.90647 (5)	0.0244 (3)	
H31	0.0416	0.7444	0.8961	0.029*	
C5	0.58482 (13)	0.11328 (10)	0.55959 (5)	0.0227 (3)	
H5	0.6489	0.1068	0.5402	0.027*	
C11	0.61224 (13)	0.60190 (10)	0.53762 (5)	0.0230 (3)	
H11	0.6716	0.5965	0.5162	0.028*	
C24	0.37914 (13)	0.46732 (10)	0.79972 (5)	0.0232 (3)	
H24	0.3456	0.5202	0.8114	0.028*	
C29	0.11984 (14)	0.11722 (9)	0.88081 (5)	0.0233 (3)	
H29	0.1817	0.1215	0.8603	0.028*	
C1	0.43502 (14)	0.04472 (10)	0.60282 (5)	0.0250 (3)	
H1	0.3971	−0.0083	0.6124	0.030*	
C26	−0.06542 (14)	0.10175 (10)	0.93967 (5)	0.0240 (3)	
H26	−0.1288	0.0952	0.9593	0.029*	
C30	0.08193 (14)	0.03369 (10)	0.89543 (5)	0.0265 (3)	
H30	0.1190	−0.0192	0.8854	0.032*	
C32	0.07329 (13)	0.61014 (9)	0.88867 (5)	0.0224 (3)	
H32	0.1301	0.6154	0.8663	0.027*	
C35	−0.09538 (13)	0.59728 (10)	0.95483 (5)	0.0231 (3)	
H35	−0.1522	0.5943	0.9773	0.028*	
C25	0.47006 (14)	0.47203 (10)	0.76910 (5)	0.0261 (3)	
H25	0.4989	0.5286	0.7601	0.031*	
C6	0.05204 (14)	0.48706 (11)	0.72970 (5)	0.0288 (3)	
H6	0.0225	0.5436	0.7384	0.035*	
C14	0.43625 (14)	0.62155 (10)	0.60020 (6)	0.0252 (3)	
H14	0.3764	0.6292	0.6213	0.030*	
C15	0.48771 (14)	0.69618 (10)	0.58015 (6)	0.0267 (3)	
H15	0.4632	0.7548	0.5877	0.032*	
C21	0.48130 (14)	0.31252 (10)	0.76462 (5)	0.0256 (3)	
H21	0.5171	0.2609	0.7526	0.031*	
C10	0.04389 (14)	0.32775 (11)	0.73544 (5)	0.0269 (3)	
H10	0.0092	0.2762	0.7480	0.032*	
H3A	0.6891 (18)	0.5011 (15)	0.6995 (7)	0.040 (5)*	
H2A	0.7816 (19)	0.3391 (13)	0.6115 (8)	0.042 (6)*	
H5A	−0.1779 (18)	0.5060 (14)	0.8017 (7)	0.042 (5)*	
H5B	−0.183 (2)	0.4236 (15)	0.8260 (8)	0.048 (6)*	
H3B	0.7020 (19)	0.4219 (14)	0.6736 (7)	0.041 (5)*	
H1A	0.761 (2)	0.1505 (14)	0.4580 (8)	0.044 (6)*	
H1B	0.784 (2)	0.2044 (17)	0.4950 (8)	0.054 (6)*	
H2B	0.780 (2)	0.2994 (16)	0.6554 (8)	0.058 (7)*	
H4A	−0.184 (2)	0.2070 (14)	0.7798 (8)	0.047 (6)*	
H4B	−0.187 (3)	0.2301 (19)	0.8215 (10)	0.081 (9)*	

### Atomic displacement parameters (Å^2^)

**Table:**

	*U*^11^	*U*^22^	*U*^33^	*U*^12^	*U*^13^	*U*^23^
Cl01	0.02249 (17)	0.01723 (17)	0.02277 (17)	−0.00011 (12)	0.00355 (13)	−0.00023 (12)
Cl02	0.02001 (16)	0.01681 (16)	0.02501 (17)	0.00094 (12)	0.00562 (13)	0.00004 (12)
Cl03	0.02599 (18)	0.02703 (19)	0.02979 (19)	−0.00023 (14)	0.00972 (14)	0.00424 (14)
Cl04	0.03331 (19)	0.02561 (19)	0.03073 (19)	0.00059 (14)	0.01765 (15)	0.00042 (14)
Cl05	0.0433 (2)	0.0277 (2)	0.02738 (19)	−0.00284 (16)	0.01019 (16)	−0.00100 (14)
Cl06	0.0475 (3)	0.0177 (2)	0.0515 (3)	0.00120 (15)	−0.0030 (2)	0.00063 (15)
O5	0.0277 (6)	0.0328 (6)	0.0302 (6)	0.0034 (5)	0.0133 (5)	0.0089 (5)
O3	0.0305 (6)	0.0310 (6)	0.0284 (6)	−0.0026 (5)	0.0117 (5)	−0.0055 (5)
O2	0.0363 (6)	0.0271 (6)	0.0311 (6)	−0.0062 (5)	0.0117 (5)	−0.0014 (5)
O1	0.0458 (7)	0.0299 (7)	0.0313 (7)	0.0061 (5)	0.0130 (6)	−0.0008 (5)
N11	0.0168 (5)	0.0166 (6)	0.0207 (6)	0.0013 (4)	0.0023 (4)	0.0007 (4)
N10	0.0193 (6)	0.0178 (6)	0.0200 (6)	−0.0003 (4)	0.0041 (4)	−0.0017 (4)
N6	0.0177 (5)	0.0182 (6)	0.0223 (6)	−0.0009 (4)	0.0040 (5)	−0.0013 (5)
N5	0.0196 (6)	0.0209 (6)	0.0200 (6)	0.0002 (5)	0.0058 (4)	−0.0012 (5)
O4	0.0417 (7)	0.0501 (8)	0.0365 (7)	0.0121 (6)	−0.0004 (6)	−0.0063 (6)
N4	0.0207 (6)	0.0205 (6)	0.0204 (6)	0.0020 (5)	0.0043 (5)	0.0019 (5)
N12	0.0197 (6)	0.0176 (6)	0.0202 (6)	0.0011 (4)	0.0044 (4)	−0.0004 (4)
N9	0.0227 (6)	0.0178 (6)	0.0277 (6)	0.0037 (5)	0.0053 (5)	−0.0045 (5)
N3	0.0235 (6)	0.0177 (6)	0.0280 (6)	−0.0024 (5)	0.0060 (5)	0.0029 (5)
N1	0.0266 (6)	0.0159 (6)	0.0241 (6)	0.0045 (5)	0.0018 (5)	−0.0009 (5)
N8	0.0291 (6)	0.0159 (6)	0.0228 (6)	−0.0031 (5)	0.0053 (5)	0.0019 (5)
N7	0.0201 (6)	0.0362 (7)	0.0204 (6)	−0.0038 (5)	0.0077 (5)	−0.0024 (5)
N2	0.0217 (6)	0.0391 (7)	0.0214 (6)	0.0031 (5)	0.0095 (5)	0.0048 (5)
C16	0.0168 (6)	0.0188 (7)	0.0182 (6)	−0.0002 (5)	0.0010 (5)	0.0011 (5)
C36	0.0170 (6)	0.0185 (7)	0.0163 (6)	−0.0004 (5)	0.0021 (5)	0.0000 (5)
C37	0.0152 (6)	0.0180 (7)	0.0157 (6)	0.0001 (5)	0.0007 (5)	−0.0001 (5)
C17	0.0183 (6)	0.0198 (7)	0.0176 (6)	0.0013 (5)	0.0021 (5)	0.0010 (5)
C38	0.0161 (6)	0.0181 (7)	0.0170 (6)	0.0000 (5)	0.0018 (5)	0.0007 (5)
C23	0.0172 (6)	0.0228 (7)	0.0171 (6)	−0.0013 (5)	0.0019 (5)	−0.0023 (5)
C18	0.0168 (6)	0.0185 (7)	0.0192 (6)	0.0002 (5)	0.0016 (5)	−0.0010 (5)
C33	0.0170 (6)	0.0169 (7)	0.0198 (6)	0.0002 (5)	0.0014 (5)	−0.0014 (5)
C28	0.0172 (6)	0.0193 (7)	0.0164 (6)	0.0010 (5)	−0.0002 (5)	−0.0002 (5)
C34	0.0208 (7)	0.0181 (7)	0.0238 (7)	−0.0007 (5)	0.0070 (5)	0.0013 (5)
C8	0.0186 (7)	0.0252 (7)	0.0171 (6)	0.0023 (6)	0.0025 (5)	0.0015 (5)
C3	0.0175 (6)	0.0195 (7)	0.0191 (6)	0.0004 (5)	−0.0002 (5)	−0.0001 (5)
C12	0.0216 (7)	0.0188 (7)	0.0236 (7)	0.0015 (5)	0.0064 (6)	−0.0017 (5)
C22	0.0217 (7)	0.0228 (7)	0.0222 (7)	−0.0015 (6)	0.0042 (6)	−0.0025 (6)
C4	0.0205 (7)	0.0196 (7)	0.0212 (7)	−0.0009 (5)	0.0031 (5)	−0.0008 (5)
C27	0.0247 (7)	0.0181 (7)	0.0224 (7)	0.0019 (6)	0.0071 (6)	−0.0002 (5)
C13	0.0178 (6)	0.0190 (7)	0.0198 (7)	−0.0007 (5)	0.0025 (5)	−0.0007 (5)
C2	0.0216 (7)	0.0228 (7)	0.0260 (7)	0.0005 (6)	0.0059 (6)	0.0025 (6)
C9	0.0234 (7)	0.0250 (8)	0.0228 (7)	0.0008 (6)	0.0046 (6)	0.0034 (6)
C7	0.0265 (7)	0.0237 (8)	0.0262 (7)	0.0024 (6)	0.0088 (6)	0.0039 (6)
C31	0.0249 (7)	0.0172 (7)	0.0317 (8)	−0.0013 (6)	0.0065 (6)	0.0014 (6)
C5	0.0216 (7)	0.0247 (7)	0.0219 (7)	0.0019 (6)	0.0036 (6)	−0.0015 (6)
C11	0.0218 (7)	0.0243 (7)	0.0240 (7)	0.0003 (6)	0.0075 (6)	−0.0008 (6)
C24	0.0240 (7)	0.0232 (7)	0.0235 (7)	−0.0024 (6)	0.0067 (6)	−0.0022 (6)
C29	0.0230 (7)	0.0202 (7)	0.0282 (8)	0.0005 (6)	0.0094 (6)	−0.0007 (6)
C1	0.0269 (7)	0.0191 (7)	0.0290 (8)	−0.0007 (6)	0.0040 (6)	0.0035 (6)
C26	0.0265 (7)	0.0238 (7)	0.0230 (7)	−0.0016 (6)	0.0083 (6)	−0.0004 (6)
C30	0.0301 (8)	0.0182 (7)	0.0328 (8)	0.0024 (6)	0.0100 (6)	−0.0027 (6)
C32	0.0222 (7)	0.0198 (7)	0.0269 (7)	−0.0009 (6)	0.0087 (6)	0.0005 (6)
C35	0.0225 (7)	0.0235 (7)	0.0246 (7)	0.0016 (6)	0.0075 (6)	−0.0008 (6)
C25	0.0269 (7)	0.0269 (8)	0.0255 (7)	−0.0051 (6)	0.0074 (6)	−0.0006 (6)
C6	0.0295 (8)	0.0309 (8)	0.0279 (8)	0.0079 (6)	0.0103 (6)	0.0009 (6)
C14	0.0253 (7)	0.0211 (7)	0.0319 (8)	0.0009 (6)	0.0129 (6)	−0.0016 (6)
C15	0.0280 (8)	0.0180 (7)	0.0360 (8)	0.0024 (6)	0.0116 (6)	−0.0019 (6)
C21	0.0230 (7)	0.0297 (8)	0.0245 (7)	0.0015 (6)	0.0053 (6)	−0.0068 (6)
C10	0.0239 (7)	0.0318 (8)	0.0261 (7)	−0.0002 (6)	0.0069 (6)	0.0066 (6)

### Geometric parameters (Å, º)

**Table:**

O5—H5A	0.92 (2)	C18—C13	1.4917 (19)
O5—H5B	0.81 (2)	C33—C32	1.3899 (19)
O3—H3A	0.84 (2)	C33—C34	1.3928 (19)
O3—H3B	0.84 (2)	C28—C27	1.3865 (19)
O2—H2A	0.86 (2)	C28—C29	1.3926 (19)
O2—H2B	0.84 (2)	C34—C35	1.378 (2)
O1—H1A	0.77 (2)	C34—H34	0.9300
O1—H1B	0.87 (2)	C8—C9	1.392 (2)
N11—C38	1.3325 (17)	C8—C7	1.393 (2)
N11—C37	1.3332 (17)	C3—C4	1.3904 (19)
N10—C36	1.3325 (17)	C3—C2	1.394 (2)
N10—C37	1.3395 (17)	C12—C11	1.377 (2)
N6—C18	1.3324 (17)	C12—C13	1.3932 (19)
N6—C16	1.3348 (17)	C12—H12	0.9300
N5—C18	1.3336 (17)	C22—C21	1.380 (2)
N5—C17	1.3350 (18)	C22—H22	0.9300
O4—H4A	0.78 (2)	C4—C5	1.378 (2)
O4—H4B	0.83 (3)	C4—H4	0.9300
N4—C17	1.3338 (18)	C27—C26	1.380 (2)
N4—C16	1.3369 (17)	C27—H27	0.9300
N12—C36	1.3355 (17)	C13—C14	1.386 (2)
N12—C38	1.3378 (17)	C2—C1	1.374 (2)
N9—C31	1.3364 (19)	C2—H2	0.9300
N9—C35	1.3387 (19)	C9—C10	1.377 (2)
N9—H09	0.8600	C9—H9	0.9300
N3—C15	1.3351 (19)	C7—C6	1.377 (2)
N3—C11	1.3359 (19)	C7—H7	0.9300
N3—H03	0.8600	C31—C32	1.377 (2)
N1—C5	1.3336 (19)	C31—H31	0.9300
N1—C1	1.3438 (19)	C5—H5	0.9300
N1—H01	0.8600	C11—H11	0.9300
N8—C26	1.3300 (19)	C24—C25	1.377 (2)
N8—C30	1.3439 (19)	C24—H24	0.9300
N8—H08	0.8600	C29—C30	1.368 (2)
N7—C21	1.334 (2)	C29—H29	0.9300
N7—C25	1.3393 (19)	C1—H1	0.9300
N7—H07	0.8600	C26—H26	0.9300
N2—C10	1.332 (2)	C30—H30	0.9300
N2—C6	1.339 (2)	C32—H32	0.9300
N2—H02	0.8600	C35—H35	0.9300
C16—C3	1.4848 (19)	C25—H25	0.9300
C36—C23	1.4880 (19)	C6—H6	0.9300
C37—C28	1.4852 (19)	C14—C15	1.375 (2)
C17—C8	1.4883 (19)	C14—H14	0.9300
C38—C33	1.4906 (18)	C15—H15	0.9300
C23—C22	1.390 (2)	C21—H21	0.9300
C23—C24	1.394 (2)	C10—H10	0.9300
			
H5A—O5—H5B	106.5 (19)	C21—C22—H22	120.4
H3A—O3—H3B	111.1 (19)	C23—C22—H22	120.4
H2A—O2—H2B	106 (2)	C5—C4—C3	119.11 (13)
H1A—O1—H1B	104 (2)	C5—C4—H4	120.4
C38—N11—C37	114.73 (11)	C3—C4—H4	120.4
C36—N10—C37	114.68 (11)	C26—C27—C28	119.19 (13)
C18—N6—C16	114.89 (11)	C26—C27—H27	120.4
C18—N5—C17	114.45 (11)	C28—C27—H27	120.4
H4A—O4—H4B	97 (2)	C14—C13—C12	119.43 (13)
C17—N4—C16	114.62 (11)	C14—C13—C18	120.95 (12)
C36—N12—C38	114.68 (11)	C12—C13—C18	119.61 (12)
C31—N9—C35	122.67 (12)	C1—C2—C3	119.19 (13)
C31—N9—H09	118.7	C1—C2—H2	120.4
C35—N9—H09	118.7	C3—C2—H2	120.4
C15—N3—C11	122.78 (12)	C10—C9—C8	119.09 (14)
C15—N3—H03	118.6	C10—C9—H9	120.5
C11—N3—H03	118.6	C8—C9—H9	120.5
C5—N1—C1	122.81 (12)	C6—C7—C8	118.64 (14)
C5—N1—H01	118.6	C6—C7—H7	120.7
C1—N1—H01	118.6	C8—C7—H7	120.7
C26—N8—C30	122.61 (12)	N9—C31—C32	120.12 (13)
C26—N8—H08	118.7	N9—C31—H31	119.9
C30—N8—H08	118.7	C32—C31—H31	119.9
C21—N7—C25	122.43 (12)	N1—C5—C4	119.79 (13)
C21—N7—H07	118.8	N1—C5—H5	120.1
C25—N7—H07	118.8	C4—C5—H5	120.1
C10—N2—C6	122.45 (12)	N3—C11—C12	119.64 (13)
C10—N2—H02	118.8	N3—C11—H11	120.2
C6—N2—H02	118.8	C12—C11—H11	120.2
N6—C16—N4	125.05 (12)	C25—C24—C23	118.58 (13)
N6—C16—C3	117.42 (12)	C25—C24—H24	120.7
N4—C16—C3	117.52 (12)	C23—C24—H24	120.7
N10—C36—N12	125.28 (12)	C30—C29—C28	119.28 (13)
N10—C36—C23	117.42 (11)	C30—C29—H29	120.4
N12—C36—C23	117.28 (11)	C28—C29—H29	120.4
N11—C37—N10	125.28 (12)	N1—C1—C2	119.62 (13)
N11—C37—C28	117.83 (11)	N1—C1—H1	120.2
N10—C37—C28	116.87 (11)	C2—C1—H1	120.2
N4—C17—N5	125.54 (12)	N8—C26—C27	119.79 (13)
N4—C17—C8	117.00 (12)	N8—C26—H26	120.1
N5—C17—C8	117.46 (12)	C27—C26—H26	120.1
N11—C38—N12	125.32 (12)	N8—C30—C29	119.81 (13)
N11—C38—C33	117.72 (11)	N8—C30—H30	120.1
N12—C38—C33	116.96 (11)	C29—C30—H30	120.1
C22—C23—C24	119.64 (13)	C31—C32—C33	118.76 (13)
C22—C23—C36	120.26 (12)	C31—C32—H32	120.6
C24—C23—C36	120.07 (12)	C33—C32—H32	120.6
N6—C18—N5	125.43 (12)	N9—C35—C34	119.68 (13)
N6—C18—C13	117.10 (11)	N9—C35—H35	120.2
N5—C18—C13	117.46 (12)	C34—C35—H35	120.2
C32—C33—C34	119.75 (12)	N7—C25—C24	120.35 (14)
C32—C33—C38	120.34 (12)	N7—C25—H25	119.8
C34—C33—C38	119.91 (12)	C24—C25—H25	119.8
C27—C28—C29	119.32 (13)	N2—C6—C7	120.29 (14)
C27—C28—C37	120.21 (12)	N2—C6—H6	119.9
C29—C28—C37	120.47 (12)	C7—C6—H6	119.9
C35—C34—C33	119.01 (13)	C15—C14—C13	119.17 (13)
C35—C34—H34	120.5	C15—C14—H14	120.4
C33—C34—H34	120.5	C13—C14—H14	120.4
C9—C8—C7	119.53 (13)	N3—C15—C14	119.86 (13)
C9—C8—C17	119.69 (12)	N3—C15—H15	120.1
C7—C8—C17	120.77 (12)	C14—C15—H15	120.1
C4—C3—C2	119.46 (13)	N7—C21—C22	119.86 (13)
C4—C3—C16	119.71 (12)	N7—C21—H21	120.1
C2—C3—C16	120.82 (12)	C22—C21—H21	120.1
C11—C12—C13	119.11 (13)	N2—C10—C9	120.00 (14)
C11—C12—H12	120.4	N2—C10—H10	120.0
C13—C12—H12	120.4	C9—C10—H10	120.0
C21—C22—C23	119.11 (13)		

### Hydrogen-bond geometry (Å, º)

**Table:**

*D*—H···*A*	*D*—H	H···*A*	*D*···*A*	*D*—H···*A*
O1—H1*A*···Cl03^i^	0.77 (2)	2.47 (2)	3.2302 (15)	171 (2)
O1—H1*B*···Cl04	0.87 (2)	2.32 (2)	3.1856 (14)	175 (2)
O2—H2*A*···Cl04	0.86 (2)	2.28 (2)	3.1124 (15)	163.2 (18)
O2—H2*B*···Cl05	0.84 (2)	2.22 (2)	3.0515 (13)	170 (2)
O3—H3*A*···Cl06^ii^	0.84 (2)	2.22 (2)	3.0379 (13)	164.5 (18)
O3—H3*B*···O2	0.84 (2)	1.91 (2)	2.7426 (17)	176 (2)
O4—H4*A*···Cl05^iii^	0.78 (2)	2.36 (2)	3.1356 (15)	172 (2)
O4—H4*B*···Cl03	0.83 (3)	2.47 (3)	3.2626 (14)	162 (3)
O5—H5*A*···Cl06	0.92 (2)	2.12 (2)	2.9973 (12)	157.4 (17)
O5—H5*B*···Cl03	0.81 (2)	2.24 (2)	3.0466 (12)	173 (2)
N1—H01···Cl01^iv^	0.86	2.24	3.0678 (12)	161
N2—H02···O5	0.86	1.77	2.5985 (16)	162
N3—H03···Cl01	0.86	2.23	3.0405 (12)	158
N7—H07···O3	0.86	1.84	2.6472 (16)	155
N8—H08···Cl02^iv^	0.86	2.25	3.0732 (12)	159
N9—H09···Cl02	0.86	2.19	3.0337 (12)	166
C1—H1···Cl03^v^	0.93	2.57	3.4995 (15)	174
C4—H4···Cl04	0.93	2.61	3.4871 (14)	157
C5—H5···O1	0.93	2.38	3.2614 (19)	158
C9—H9···Cl06^v^	0.93	2.70	3.4071 (15)	134
C10—H10···O4	0.93	2.54	3.370 (2)	148
C11—H11···Cl02^vi^	0.93	2.61	3.5114 (15)	163
C12—H12···Cl04	0.93	2.64	3.4992 (15)	154
C15—H15···Cl03^vii^	0.93	2.54	3.3157 (16)	141
C21—H21···Cl05	0.93	2.74	3.5424 (15)	146
C22—H22···Cl06^v^	0.93	2.72	3.3883 (15)	130
C24—H24···Cl05^viii^	0.93	2.63	3.4519 (15)	147
C26—H26···Cl04^ix^	0.93	2.53	3.3669 (15)	149
C30—H30···O2^x^	0.93	2.31	3.2331 (19)	174
C31—H31···O2^viii^	0.93	2.50	3.3320 (19)	149
C34—H34···O1^ix^	0.93	2.50	3.3706 (19)	156
C35—H35···Cl01^xi^	0.93	2.72	3.6254 (14)	166

Symmetry codes: (i) *x*+1, −*y*+1/2, *z*−1/2; (ii) *x*+1, *y*, *z*; (iii) *x*−1, *y*, *z*; (iv) *x*, *y*−1, *z*; (v) −*x*, *y*−1/2, −*z*+3/2; (vi) *x*+1, −*y*+3/2, *z*−1/2; (vii) −*x*, *y*+1/2, −*z*+3/2; (viii) −*x*+1, *y*+1/2, −*z*+3/2; (ix) *x*−1, −*y*+1/2, *z*+1/2; (x) −*x*+1, *y*−1/2, −*z*+3/2; (xi) *x*−1, −*y*+3/2, *z*+1/2.
