# Supplementary figures and images for: Crystal structure of 4,4′,4′′-(1,3,5-triazine-2,4,6-tri­yl)tripyridinium trichloride 2.5-hydrate
Source: Acta Crystallogr E Crystallogr Commun. 2015 Oct 17;71(Pt 11):o858–9. doi: 10.1107/S2056989015018125 (PMC4645061; doi:10.1107/S2056989015018125)

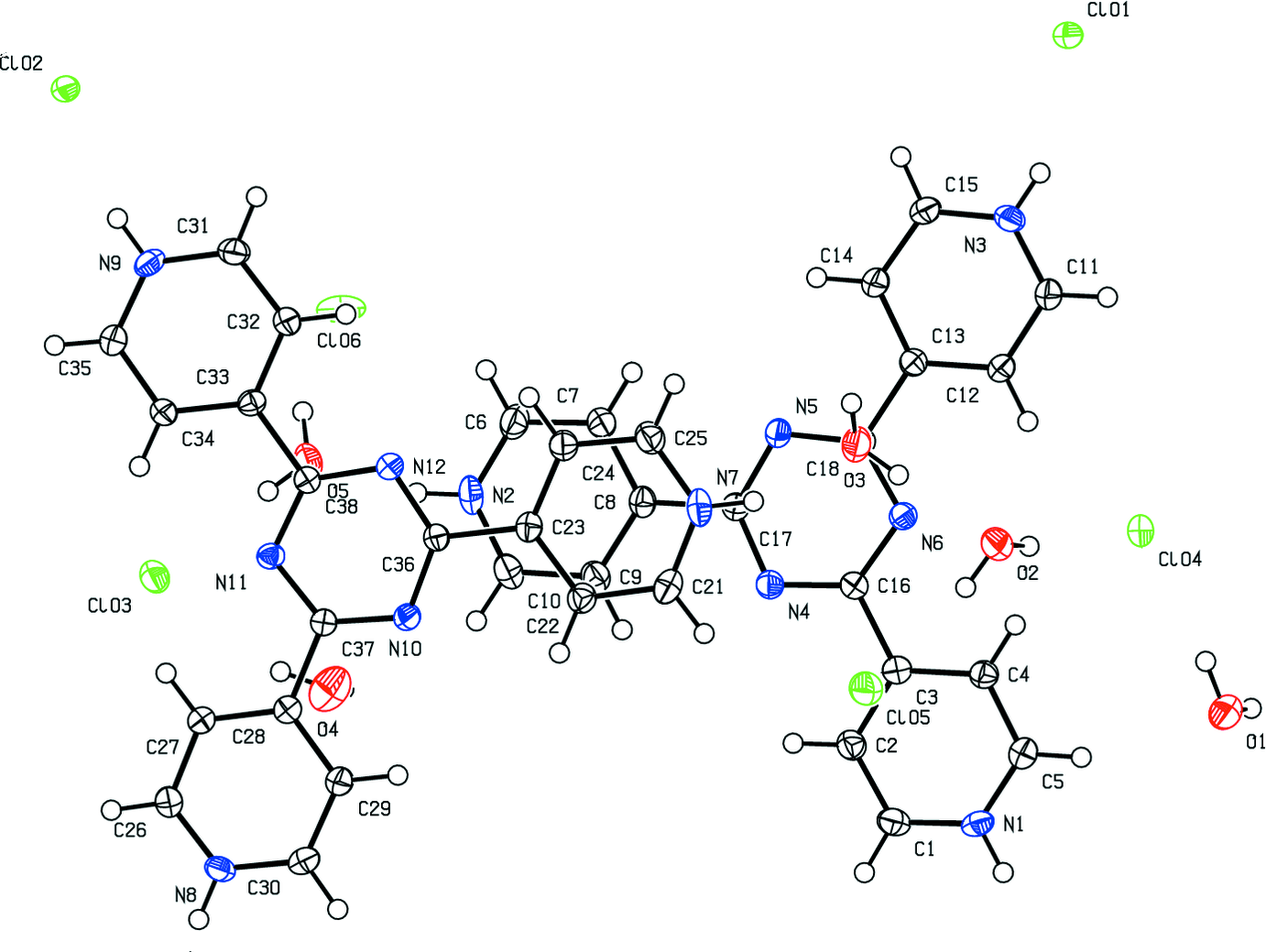

Supplement: Supplementary file 4 [file e-71-0o858-fig1.tif]

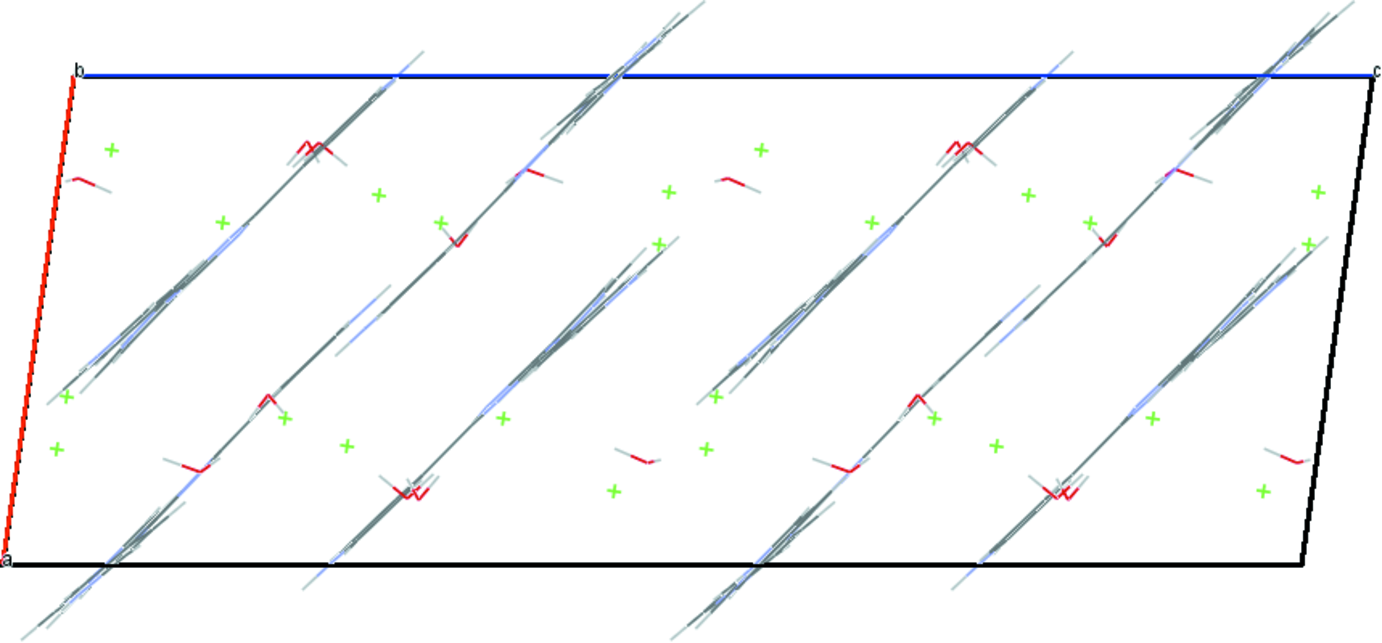

Supplement: Supplementary file 5 [file e-71-0o858-fig2.tif]
